# An environmental pollutant, 9,10‐phenanthrenequinone, activates human TRPA1 via critical cysteines 621 and 665

**DOI:** 10.1002/prp2.342

**Published:** 2017-07-28

**Authors:** Katsuhiko Muraki, Takashi Sekine, Yuna Ando, Hiroka Suzuki, Noriyuki Hatano, Tadashi Hirata, Yukiko Muraki

**Affiliations:** ^1^ Laboratory of Cellular Pharmacology School of Pharmacy Aichi‐Gakuin University 1‐100 Kusumoto, Chikusa Nagoya 464‐8650 Japan; ^2^ R&D Group Tobacco Business Headquarters Scientific Product Assessment Center Japan Tobacco Inc. Yokohama 227‐8512 Japan

**Keywords:** 9,10‐phenanthrenequinone, calcium channel, environmental pollutants, transient receptor potential

## Abstract

Transient receptor potential ankyrin 1 (TRPA1) is activated by noxious cold, mechanical stimulation, and irritant chemicals. In our recent study, 9, 10‐phenanthrenequinone (9,10‐PQ) is the most potent irritant for activation of NRF2 among 1395 cigarette smoke components and it may be, therefore, important to find its additional targets. Here, we show that 9,10‐PQ functions as an activator of TRPA1 in human embryonic kidney (HEK) cells expressing human wild‐type TRPA1 (HEK‐wTRPA1) and human alveolar A549 (A549) cells. Application of 9,10‐PQ at 0.1–10 *μ*mol/L induced a concentration‐dependent Ca^2+^ response as well as inward currents at −50 mV in HEK‐wTRPA1 cells. The current response was blocked by TRPA1 antagonists, HC‐030031 (HC) and A‐967079. To test whether 9,10‐PQ affects the cysteine residues of TRPA1, we expressed mutant TRPA1 channels in HEK cells (HEK‐muTRPA1) in which six different cysteine residues were replaced with serine. Among them, a mutation of cysteine 621 (C621S) abolished the 9,10‐PQ‐induced Ca^2+^ and current responses. The channel activity induced by 9,10‐PQ was also abolished in excised inside‐out patches isolated from HEK‐muTRPA1 cells with the C621S substitution. Although a mutation of cysteine 665 (C665S) reduced the 9,10‐PQ‐induced response, channel sensitization by pretreatment with Cu^2+^ plus 1,10‐phenanthroline and by internal dialysis of 3 *μ*mol/L Ca^2+^ restored the response. However, a double mutant with C621S and C665S substitutions had little response to 9,10‐PQ, even when sensitized by Ca^2+^ dialysis. In A549 cells, 9,10‐PQ induced an HC‐sensitive Ca^2+^ response. Our findings demonstrate that 9,10‐PQ activation of human TRA1 is dependent on cysteine residues 621 and 665.

**Abbreviations**
1,10P‐5,6‐D: 1,10‐phenanthroline‐5,6‐dione
1,10‐Pher: 1,10‐phenanthroline
1,2‐NQ: 1,2‐naphthoquinone
9,10‐PQ: 9,10‐phenanthrenequinone
A549: human alveolar A549
A96: A‐967079
AITC: allyl isothiocyanate
CSE: cigarette smoke extracts
DEP: diesel exhaust particles
EGTA: ethylene glycol tetraacetic acid
HC: HC‐030031
HEK: human embryonic kidney
HEK‐wTRPA1: HEK cells expressing human wild‐type TRPA1
Men: menthol
NRF2: nuclear factor erythroid 2‐related factor 2
P450: cytochrome P450
Phenan: phenanthrene
TBS: Tris‐buffered saline
TPP: tripolyphosphate
TRPA1: transient receptor potential ankyrin 1
WSPM: wood smoke particulate material

## Introduction

A chemical compound, 9,10‐PQ, which is one of the active components in cigarette smoke and other environmental pollutants, affects a number of mammalian physiological functions via activation of a transcription factor, nuclear factor erythroid 2‐related factor 2 (NRF2), production of hydrogen peroxide, and/or the covalent modification of cysteine residues of proteins (Kishikawa et al. 2010; Asahi et al. 2014; Sekine et al. 2016). Treatment of cells with 9,10‐PQ, therefore, causes nonacute biological responses such as antiproliferation, inflammation, and apoptosis (Matsunaga et al. 2010, 2012; Hatae et al. 2013). Our recent study has revealed that 9,10‐PQ is the most potent chemical to activate NRF2 among 1395 mainstream cigarette smoke components (Sekine et al. 2016), suggesting it can be an exogenous bioactive compound that, when inhaled, has multiple potential targets, particularly in the acute phase of the exposure. In particular, 9,10‐PQ is 12.6 times more potent than acrolein, which also effectively activates a cationic channel protein, TRPA1 (Andre et al. 2008).

TRPA1, which is activated by noxious cold, mechanical stress, irritant chemicals, and/or some clinical drugs, is widely expressed in neuronal and non‐neuronal organs (Story et al. 2003; Jordt et al. 2004; Macpherson et al. 2007; Fajardo et al. 2008; Matta et al. 2008; Nassini et al. 2010; Andersson et al. 2011; Hatano et al. 2013). Transgenic mice lacking TRPA1 are less sensitive to mechanical stimulation, cold stimuli, and tumor necrosis factor *α*‐induced mechanical hyperalgesia, which suggests that TRPA1 is a nociceptor that mediates acute and inflammatory pain (Obata et al. 2005; Bautista et al. 2006; Kwan et al. 2006; Karashima et al. 2009; del Camino et al. 2010; Fernandes et al. 2011). On the other hand, several cysteine residues of TRPA1 are targeted by physiological and nonphysiological electrophilic compounds that activate the channel (Hinman et al. 2006; Macpherson et al. 2007). In particular, a recent study revealed that a cysteine residue at 621 (C621) is critical for channel activation by the electrophilic compounds, and that cysteine 665 (C665) is not only supportive but also important for its activation (Bahia et al. 2016). In contrast, nonelectrophilic compounds such as Δ9‐tetrahydrocannabinol, nicotine, and menthol (Men) activate TRPA1 via different mechanisms (Jordt et al. 2004; Karashima et al. 2007; Talavera et al. 2009). Since numerous structurally unrelated compounds stimulate TRPA1, research should focus on whether the channel is activated by clinical drugs and environmental chemicals.

In the current study, we show that a chemical pollutant, 9,10‐PQ, functions as an activator of human TRPA1. A comparison of the 9,10‐PQ‐induced responses of wild‐type and mutant TRPA1 with responses to allyl isothiocyanate (AITC) identified that both C621 and C665 are important for the 9,10‐PQ‐induced response, but that C621 is most critical for activation. Treatment with 9,10‐PQ also effectively activated endogenous TRPA1 in human alveolar A549 cells. Our findings indicate that 9,10‐PQ directly activates human TRPA1 via interaction with C621 and C665.

## Materials and Methods

### Cell culture

Human embryonic kidney (HEK) cells obtained from the Health Science Research Resources Bank (HSRRB, Osaka, Japan) were maintained in Dulbecco's modified minimum essential medium (D‐MEM; Sigma‐Aldrich, St. Louis, MO) supplemented with 10% heat‐inactivated fetal calf serum (FCS; Sigma‐Aldrich), penicillin G (100 U/mL), and streptomycin (100 *μ*g/mL). Human alveolar A549 cells (A549 cells, DS Pharma Biomedical Co., Osaka Japan) were cultured in D‐MEM containing 10% FCS, 100 U/mL penicillin G, and 100 *μ*g/mL streptomycin, as recommended by the supplier.

### Recombinant expression of TRP channels in HEK cells

Partially confluent HEK cells (40–60% confluency) were transfected with the pcDNA3.1 and pcAc‐GFP plasmids containing human TRPA1 and TRPV1 cloned by ourselves, respectively, using lipofectamine 3000 (Thermo Fisher Scientific, Yokohama, Japan). The TRPA1 mutations were constructed by PCR using mutant oligonucleotide primers in which the cysteine residues at amino acid positions 414, 421, 621, 633, 641, or 665 were replaced with serine (Agilent Technologies, Santa Clara, CA), and all constructs were sequenced. All experiments were performed within 48 h of transfection.

### Western blotting

HEK cells were lysed in 50 *μ*L lysis buffer ([in mmol/L] Tris‐HCl 50 [pH 8.0], NaCl 150, EDTA 5) including 1% NP‐40, 0.5% sodium deoxycholate, 0.1% SDS, and protease inhibitors (Suzuki et al. 2014). The cell lysates were incubated on ice for 30 min, vortexed every 5 min, then centrifuged at 20,000*g* for 30 min at 4°C, and each lysate (10 *μ*g protein) was separated on an 8% polyacrylamide gel. Proteins were then transferred to a PVDF membrane. After blocking nonspecific antibodies for 2 h in Tris‐buffered saline (TBS) containing 5% skim milk and 0.1% Tween‐20, the PVDF membrane was incubated with the primary antibody (goat anti‐human TRPA1; Santa Cruz Biotechnology Inc., Dallas, TX; 1:1000 dilution) overnight at 4°C. The membrane was then washed three times with washing buffer (TBS containing 0.1% Tween‐20) and exposed to the secondary antibody (IgG‐HRP; 1:10,000 dilution). The membrane was washed again, and detection reagents (Merck Millipore, Darmstadt, Germany) were added to generate a chemiluminescence signal. To determine the relative quantity of TRPA1 to *β*‐actin in each sample the membrane was also exposed to a mouse anti‐*β*‐actin monoclonal antibody (1:2000 dilution). Finally, detection was carried out using an LAS‐3000 mini apparatus (Fujifilm, Tokyo, Japan).

### Patch clamp experiments

Whole‐cell and single‐channel recording experiments were performed as described previously (Suzuki et al. 2014). The resistance of electrodes was 3–5 MΩ when filled with pipette solution. A Cs^+^‐rich pipette solution for whole‐cell recordings contained (in mM) Cs‐aspartate 110, CsCl 30, MgCl_2_ 1, HEPES 10, and Na_2_ATP 2 (adjusted to pH 7.2 with CsOH). The free Ca^2+^ concentration in the pipette solution was adjusted to 0 (1 mmol/L ethylene glycol tetraacetic acid; EGTA), 0.3 (10 mmol/L EGTA and 6.25 mmol/L CaCl_2_), and 3 *μ*mol/L (10 mmol/L EGTA and 9.51 mmol/L CaCl_2_) using Ca^2+^‐EGTA buffer. When tripolyphosphate (TPP) was used to maintain TRPA1 channel activity, a bathing or a pipette solution contained (in mmol/L) CsCl 140, MgCl_2_ 1, EGTA 1, HEPES 10, and TPP 2.89 (adjusted to pH 7.2 with CsOH). Membrane currents and voltage signals were digitized using an analogue‐to‐digital converter (PCI6229, National Instruments Japan, Tokyo, Japan) driven by WinEDRV3.38 for data acquisition and analysis of whole‐cell currents and excised outside‐out and inside‐out single‐channel currents (developed by Dr. John Dempster, University of Strathclyde, UK). The liquid junction potential between the pipette and bath solutions (−10 mV) was corrected when aspartate^−^‐rich pipette solution was used. A ramp voltage protocol from −150 mV to +50 mV for 100 ms was applied every 5 sec from a holding potential of −50 mV. A leak current component was not subtracted from the recorded currents. The outside‐out and inside‐out patch channel activity was estimated to calculate NPOs, where N is the number of channels in the patch and PO is the open probability. A standard HEPES‐buffered bathing solution (2.2 Ca SBS [in mmol/L]: NaCl 137, KCl 5.9, CaCl_2_ 2.2, MgCl_2_ 1.2, glucose 14, HEPES 10 [adjusted to pH 7.4 with NaOH]) was used. When CaCl_2_ was omitted from the 2.2 Ca SBS (0 Ca SBS), 10 mmol/L CsCl was added. All experiments were performed at 25 ± 1°C.

### Measurement of Ca^2+^ fluorescence ratio

HEK and A549 cells, which were loaded with 10 *μ*mol/L Fura2‐AM (Dojindo, Kumamoto, Japan) in the 2.2 Ca SBS for 30 min at room temperature, were superfused with 2.2 Ca SBS for 10 min and then Fura‐2 fluorescence signals were measured at 0.1 Hz using the Argus/HisCa imaging system (Hamamatsu Photonics, Hamamatsu, Japan) driven by Imagework Bench 6.0 (INDEC Medical Systems, Santa Clara, CA). Since the efficacy of gene transfection in HEK cells and the TRPA1 expression level in A549 cells was similar but not identical from cell to cell, we collected 50 and 12–38 single cells on one coverslip for analysis in HEK and A549 cells, respectively, and repeated the same experiment with the other coverslips to reduce variation. In each analysis, a whole‐cell area was chosen as a region of interest to average the fluorescence ratio.

### Data analysis

Data are expressed as the mean ± SEM. Statistical significance between two groups and among multiple groups was examined using paired or unpaired Student's *t*‐tests, and ANOVA or Tukey's test, respectively.

### Reagents

The following drugs were used: 9,10‐phenanthrenequinone (9,10‐PQ; Sigma‐Aldrich), allyl isothiocyanate (AITC; Kanto Chemical Co., Tokyo, Japan), menthol (Men, Wako Chemical Co., Osaka, Japan), ZnSO_4_ (Zn^2+^, Wako Chemical Co.), 1,10‐phenanthroline‐5,6‐dione (1,10‐P‐5,6‐D, Sigma‐Aldrich), phenanthrene (Phenan, Sigma‐Aldrich), 1,10‐phenanthroline (1,10‐Pher, Tokyo Kasei, Tokyo, Japan), HC‐030031 (HC; Enzo Life Sciences, Farmingdale, NY), A‐967079 (A96; FOCUS Biomolecules, Plymouth Meeting, PA), capsaicin (Sigma‐Aldrich), tripolyphosphate (TPP, Wako Chemical Co.), and CuSO_4_ (Cu^2+^, Sigma‐Aldrich). Each drug was dissolved in the vehicle as recommended by the manufacturer.

## Results

**Figure 1:**
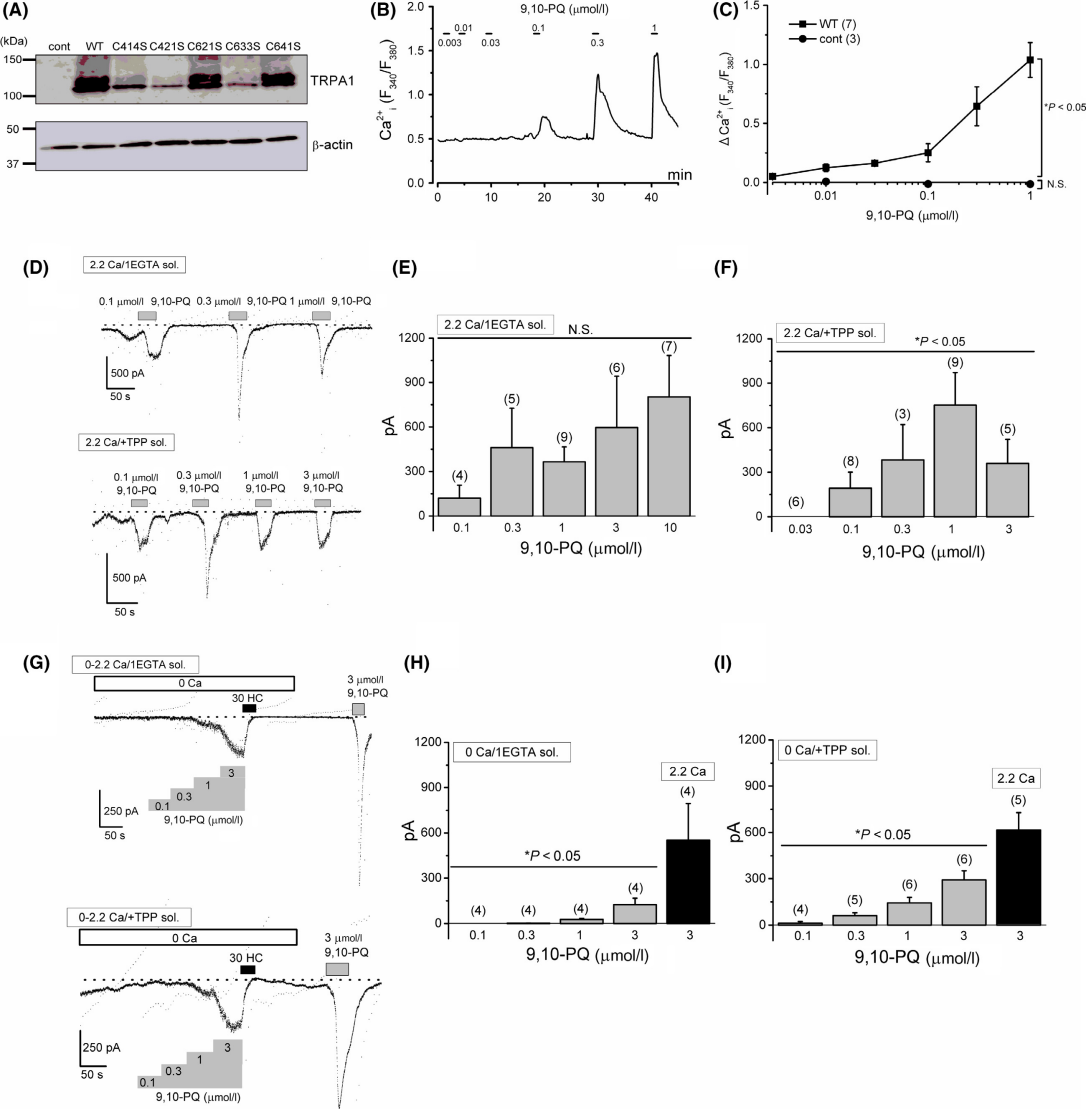
Activation of TRPA1 by 9,10‐PQ in HEK‐wTRPA1 cells. (A) Wild‐type (WT) and mutant TRPA1 proteins (C414S, C421S, C621S, C633S, and C641S) were assayed by western blotting to confirm the protein expression in HEK cells. *β*‐actin levels were assayed as controls. 9,10‐PQ induced Ca^2+^ responses in a representative HEK‐wTRPA1 cell (B) and the peak 9,10‐PQ‐induced Ca^2+^ response (ΔCa^2+^_i_ [F_340_/F_380_]) in HEK‐wTRPA1 cells (WT, seven independent experiments) and control HEK cells (cont, three independent experiments) was summarized as a concentration–response relationship (C). *P <* *0.05 by ANOVA. 9,10‐PQ‐induced membrane currents at −50 mV in a representative HEK‐wTRPA1 cell which was superfused with 1 mmol/L EGTA (the upper trace) and 1 mmol/L EGTA plus 2.89 mmol/L TPP (the lower trace) (D) and the peak 9,10‐PQ‐induced inward currents at −50 mV was summarized in HEK‐wTRPA1 cells as a concentration–response relationship (4–9 and 3–9 independent experiments, respectively, in (E) and (F)). Cells were superfused with 2.2 Ca SBS as a bathing solution. Ramp waveform voltage pulses from −150 to +50 mV for 100 msec, shown by vertical dotted lines, were applied every 5 sec. (G–I) The experimental conditions were identical to those demonstrated in (D–F), except superfusion contained 0 Ca SBS and addition of 30 *μ*mol/L HC after cumulative application of 9,10‐PQ. At the end of each experiment, 3 *μ*mol/L 9,10‐PQ was applied again in 2.2 Ca SBS (4 and 4–6 independent experiments, respectively, in (H) and (I)). *P <* *0.05 by ANOVA. Bars = SEM. N.S. shows no significance.

Expression of wild‐type and mutant TRPA1 in HEK cells was confirmed using the following three assays as described previously (Suzuki et al. 2014): TRPA1 mRNA transcript expression (not shown), TRPA1 protein expression (Fig. 1A), and TRPA1 channel function (Figs. 1B–I, Figs. 4–8). Using HEK‐wTRPA1 cells, we examined the effects of 9,10‐PQ on TRPA1. As shown in Figure 1B and C, treatment of HEK‐wTRPA1 cells with 9,10‐PQ at concentrations between 0.003 and 1 *μ*mol/L effectively induced Ca^2+^ responses, whereas failed to evoke any Ca^2+^ response in control HEK cells (cont, Fig. 1C). To confirm that 9,10‐PQ activates TRPA1 channel currents, we applied 0.1–10 *μ*mol/L 9,10‐PQ to HEK‐wTRPA1 cells superfused with 2.2 Ca SBS in the whole‐cell recording mode. As shown in the upper panel of Figure 1D and also in Figure 1E, the exposure to 9,10‐PQ induced inward currents at −50 mV, which had a dose‐independent variable size and were transient even in the presence of 9,10‐PQ. When 2.89 mmol/L TPP, a stabilizer of TRPA1 (Kim and Cavanaugh 2007; Suzuki et al. 2014), was included in the pipette solution, application of 9,10‐PQ significantly induced inward currents at −50 mV, which were still variable and sometimes transient (lower panel of Fig. 1D and F). In contrast, the 9,10‐PQ‐induced currents became sustainable upon the removal of extracellular Ca^2+^ (0 SBS), although the current size was smaller (Fig. 1G and H). In the presence of TPP in the pipette solution, the 9,10‐PQ‐induced inward currents were larger and dose dependent (Fig. 1I). Moreover, these 9,10‐PQ‐induced currents were abolished with the addition of 30 *μ*mol/L HC, a TRPA1 antagonist, suggesting that 9,10‐PQ can activate human TRPA1.

**Figure 2:**
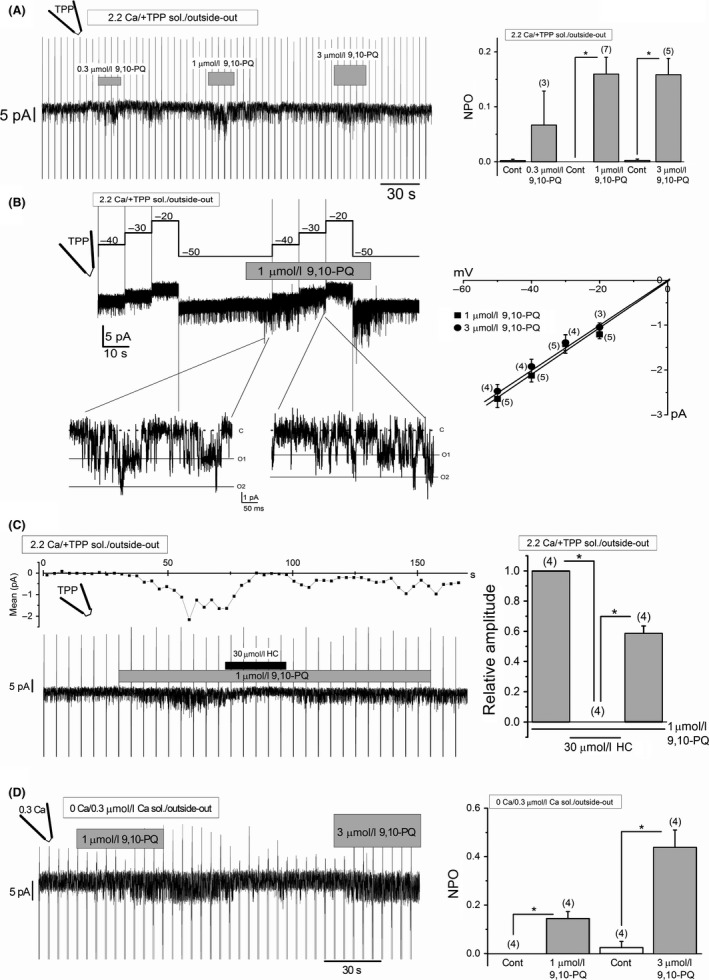
Activation of TRPA1 channels by 9,10‐PQ in excised outside‐out patches. (A) A representative 9,10‐PQ‐evoked single‐channel current trace at −50 mV in a patch excised from a HEK‐wTRPA1 cell (left panel) and the summary of channel activity of NPO (0.3–3 *μ*mol/L 9,10‐PQ, 3–7 independent experiments) (right panel). Each patch was superfused with 2.2 Ca SBS and dialyzed with CsCl‐rich pipette solution including 2.89 mmol/L TPP. Vertical lines indicate application of ramp pulses. *P <* *0.05 by paired Student's *t*‐test. (B) A representative 9,10‐PQ‐evoked single‐channel current trace by a stepwise change of voltage from −50 mV to −20 mV in a patch excised from a HEK‐wTRPA1 cell (left panel) and a summary of the relationship between the current and voltage of 1 and 3 *μ*mol/L 9,10‐PQ‐evoked single‐channel currents (3–5 independent experiments) (right panel). In the lower panel of the trace (B), time and current scales were expanded. Each patch was superfused with 2.2 Ca SBS and dialyzed with CsCl‐rich pipette solution including 2.89 mmol/L TPP. The “c” indicates the channel closing level, and “O1” and “O2” represent the first and second channel opening levels, respectively. (C) Inhibitory effects of HC on 9,10‐PQ‐evoked TRPA1 channel activity in excised outside‐out patches. An original current trace (lower panel) and averaged current amplitude for every 3 sec (upper panel) are shown at the left. While 1 *μ*mol/L 9,10‐PQ was present in 2.2 Ca SBS, 30 *μ*mol/L HC was added. The relative current amplitude before and during HC application, and after washout, is summarized in the right panel (four independent experiments). Each patch was superfused with 2.2 Ca SBS and dialyzed with CsCl‐rich pipette solution including 2.89 mmol/L TPP. *P <* *0.05 by Tukey's test. (D) A representative 9,10‐PQ‐evoked single‐channel current trace at −50 mV in a patch excised from a HEK‐wTRPA1 cell and the summary of the channel activity of NPO (four independent experiments). Each patch was superfused with 0 Ca SBS and dialyzed with Cs‐aspartate‐rich pipette solution including 0.3 *μ*mol/L Ca^2+^. Vertical lines indicate application of ramp pulses. *P <* *0.05 by paired Student's *t*‐test. Bars = SEM.

In intact cells, ion channels might be influenced by biologically active factors, which are mainly produced intracellularly. In addition, 9,10‐PQ can cause oxidative stress that changes cellular signaling pathways. To reduce the possibility that such factors might be involved in 9,10‐PQ‐induced channel activation of TRPA1, we next examined whether 9,10‐PQ directly activates TRPA1 channels in outside‐out patches excised from HEK‐wTRPA1 cells. As shown in Figure 2A, application of 0.3–3 *μ*mol/L 9,10‐PQ also induced channel activity in the excised patches. The channel conductance at negative potentials was 50.1 ± 4.4 pS (*n* = 5) and 49.6 ± 3.2 pS (*n* = 4) in the presence of 1 and 3 *μ*mol/L 9,10‐PQ, respectively (Fig. 2B), and the unit current amplitude at −50 mV (−2.64 ± 0.19 pA and −2.47 ± 0.15 pA by 1 and 3 *μ*mol/L 9,10‐PQ, see also Fig. 2B) was similar to that evoked by 30 *μ*mol/L AITC (−2.64 ± 0.28 pA, *n* = 5) under experimental conditions that were similar to our previous study (Suzuki et al. 2014). Moreover, the channel activity was reversibly inhibited by the addition of HC (Fig. 2C), suggesting that TRPA1 is activated by 9,10‐PQ in a membrane‐delimited manner. For comparison, we also examined the effects of 9,10‐PQ on channel activity in excised outside‐out patches where 0.3 *μ*mol/L Ca^2+^ was included in the pipette solution without TPP in 0 Ca SBS (Fig. 2D); application of 9,10‐PQ at 1 and 3 *μ*mol/L effectively induced channel activity.

**Figure 3:**
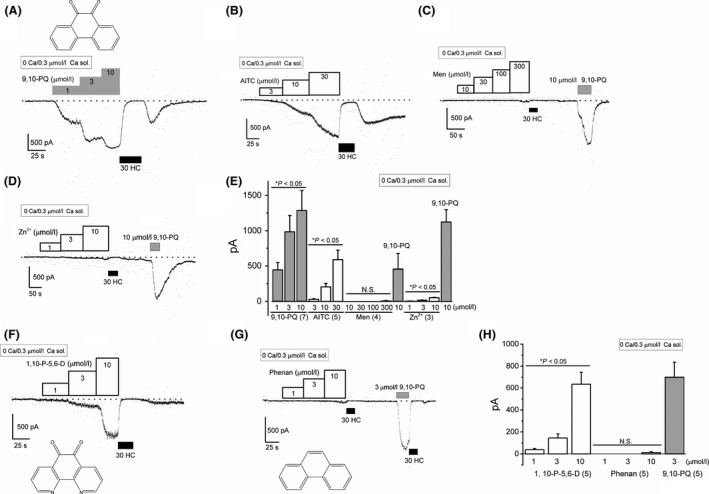
Comparison of TRPA1 channel activation by 9,10‐PQ with conventional TRPA1 agonists and structurally related compounds. Cells were superfused with 0 Ca SBS and dialyzed with Cs‐aspartate‐rich pipette solution containing 0.3 *μ*mol/L Ca^2+^. Ramp waveform voltage pulses from −150 to +50 mV for 100 msec were applied every 5 sec. Effects of 9,10‐PQ (A, 1–10 *μ*mol/L), AITC (B, 3–30 *μ*mol/L), Men (C, 10–300 *μ*mol/L), and Zn^2+^ (D, 1–10 *μ*mol/L) on membrane currents at −50 mV in each HEK‐wTRPA1 cell. After the final concentration of each agonist was applied, 30 *μ*mol/L HC (black rectangles) was added to block TRPA1 channel current components. As a comparison, 10 *μ*mol/L 9,10‐PQ was applied in (C) and (D). The chemical structure of 9,10‐PQ is illustrated in the inset of (A). (E) The peak agonist‐induced inward currents at −50 mV were pooled and summarized as a concentration–response relationship (3–7 independent experiments). The *x*‐axis indicates concentration of each compound. *P <* *0.05 by ANOVA. (F–H) The experimental conditions were identical to those demonstrated in (A–E) except for the application of 1–10 *μ*mol/L 1,10‐P‐5,6‐D (five independent experiments) (F) and 1–10 *μ*mol/L Phenan (five independent experiments) (G). Chemical structures of 1,10‐P‐5,6‐D and Phenan are illustrated in the insets of (F) and (G), respectively. (H) The peak compound‐induced inward currents at −50 mV were pooled and summarized as a concentration–response relationship. The *x*‐axis indicates concentration of each compound. *P <* *0.05 by ANOVA. N.S. shows no significance. Bars = SEM.

It has been widely shown that electrophilic and nonelectrophilic compounds can activate TRPA1 (Nilius et al. 2011). We, therefore, compared agonistic activity against TRPA1 among 9,10‐PQ, AITC, Men, and Zn^2+^ (Fig. 3). To obtain relatively large and sustainable TRPA1 channel currents during the recording, we filled the pipette with a Cs‐aspartate‐rich solution including 0.3 *μ*mol/L Ca^2+^. Although cumulative application of 9,10‐PQ and AITC induced relatively large HC‐sensitive inward currents at −50 mV (Fig. 3A, B, and E), Zn^2+^ induced only small inward currents at −50 mV (Fig. 3D and E). In contrast, at a concentration between 10 and 300 *μ*mol/L, Men did not induce any inward currents (Fig. 3C and E). The quinone moiety of 9,10‐PQ is potentially important for the activation of TRPA1 as several other quinone‐related compounds also cause TRPA1 activation due to their high electrophilicity (Ibarra and Blair 2013). To confirm this possibility, we next examined the effects of 1,10‐phenanthroline‐5,6‐dione (1,10‐P‐5,6‐D) and phenanthrene (Phenan) with and without the quinone moiety, respectively, on TRPA1 (Fig. 3F and G). Application of 1–10 *μ*mol/L 1,10‐P‐5,6‐D induced smaller inward currents at −50 mV than 9,10‐PQ, which were abolished by HC (Fig. 3F and H), whereas Phenan at a concentration between 1 and 10 *μ*mol/L did not induce TRPA1 channel currents (Fig. 3G and H). Moreover, 1,10‐phenanthroline (1,10‐Pher, 15 *μ*mol/L) in the absence of the quinone moiety of 1,10‐P‐5,6‐D failed to induce TRPA1 channel currents at −50 mV (0 pA by 1,10‐Pher vs. 489.8 ± 138 pA by 1,10‐Pher plus 3 *μ*mol/L 9,10‐PQ, *P* < 0.05, *n* = 5), suggesting that the quinone moiety of 9,10‐PQ plays an obligatory role in activation of human TRPA1.

**Figure 4:**
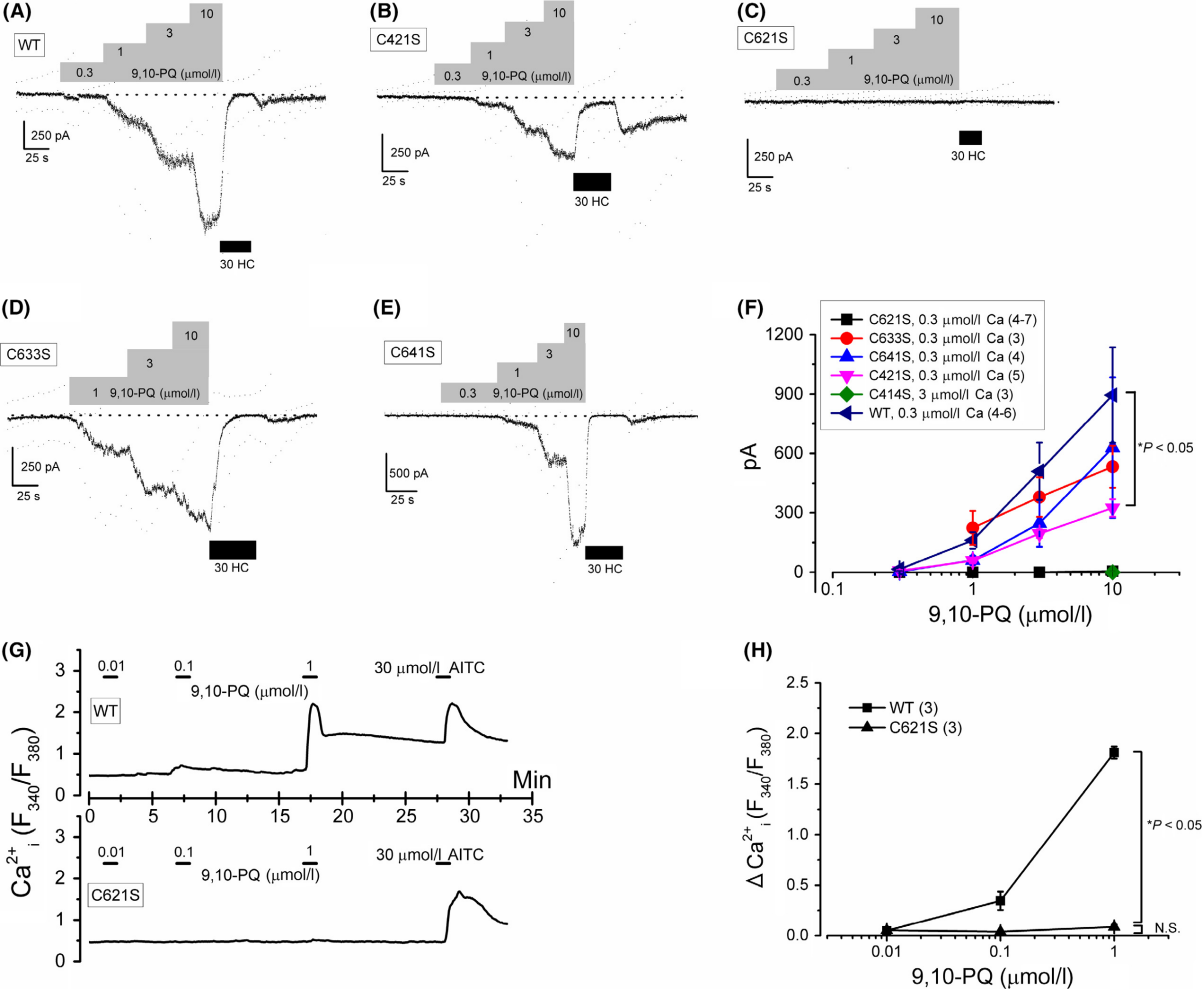
Comparison of 9,10‐PQ‐induced TRPA1 channel activation among mutants in TRPA1 N‐terminal cysteine residues. Cells in which a cysteine residue of wild TRPA1 (WT, A) was mutated with serine at 421 (C421S, B), 621 (C621S, C), 633 (C633S, D), and 641 (C641S, E) were superfused with 0 Ca SBS and dialyzed with Cs‐aspartate‐rich pipette solution including 0.3 *μ*mol/L Ca^2+^. Ramp waveform voltage pulses from −150 to +50 mV for 100 msec were applied every 5 sec. 9,10‐PQ was commutatively applied to each mutant to confirm effects on membrane currents at −50 mV. After application of the final concentration of 9,10‐PQ, 30 *μ*mol/L HC (black rectangles) was added to block TRPA1 channel current components. (F) The peak 9,10‐PQ‐induced inward currents at −50 mV were pooled and summarized as a concentration–response relationship (3–7 independent experiments). In HEK‐muTRPA1 cells with a C414S substitution, 3 *μ*mol/L Ca^2+^ was intracellularly dialyzed to potentiate TRPA1 channel currents. *P <* *0.05 by Tukey's test in the presence of 10 *μ*mol/L 9,10‐PQ (C621S vs. WT, C421S, C633S, and C641S). (G) and (H) Ca^2+^ responses of mutant TRPA1 with C621S to 9,10‐PQ. A representative 9,10‐PQ‐induced Ca^2+^ response was shown in a HEK‐wTRPA1 (G, upper panel) and a HEK‐muTRPA1 with C621S cell (G, lower panel). The peak 9,10‐PQ‐induced Ca^2+^ response (ΔCa^2+^_i_ [F_340_/F_380_]) was summarized as a concentration–response relationship for WT TRPA1 (3 independent experiments) and mutant TRPA1 with C621S (3 independent experiments). To confirm the channel expression, 30 *μ*mol/L AITC was applied at the end of experiment. *P <* *0.05 by ANOVA. Bars = SEM.

**Figure 5:**
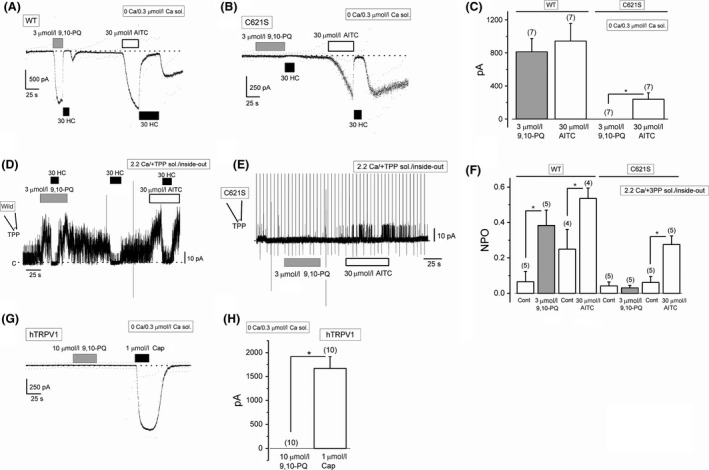
Critical importance of C621 for 9,10‐PQ‐induced TRPA1 channel response. 9,10‐PQ (3 *μ*mol/L)‐ and AITC (30 *μ*mol/L)‐induced inward currents at −50 mV were compared between HEK‐wTRPA1 (WT) (A) and HEK‐muTRPA1 cells with a C621S substitution (C621S) (B), and summarized (C, seven independent experiments each). Following agonist treatment, 30 *μ*mol/L HC (black rectangles) was added to block TRPA1 channel current components. *P <* *0.05 by paired Student's *t*‐test. (D–F) 9,10‐PQ (3 *μ*mol/L)‐ and AITC (30 *μ*mol/L)‐induced single‐channel activity in excised inside‐out patches recorded from HEK‐wTRPA1 (WT) (D) and HEK‐muTRPA1 cells with a C621S substitution (C621S) (E) and the summary of NPO (F, 4–5 independent experiments). Each patch was superfused with a bathing solution containing CsCl‐rich bathing solution with 2.89 mmol/L TPP. The pipette solution included 2.2 Ca SBS. Vertical lines indicate application of ramp pulses. *P <* *0.05 by paired Student's *t*‐test. (G–H) No effects of 9,10‐PQ (10 *μ*mol/L) on human TRPV1 were shown in HEK cells expressing human TRPV1 (G) and the pooled data were summarized (H, 10 independent experiments). Cells were voltage‐clamped at −50 mV. To confirm the expression of TRPV1, 1 *μ*mol/L capsaicin (Cap) was applied at the end of each experiment. Cells were superfused with 0 Ca SBS and dialyzed with Cs‐aspartate‐rich pipette solution including 0.3 *μ*mol/L Ca^2+^. Ramp waveform voltage pulses from −150 to +50 mV for 100 msec were applied every 5 sec. *P <* *0.05 by paired Student's *t*‐test. Bars = SEM.

The conservative cysteine‐to‐serine mutations in the predicted N‐terminal ankyrin repeats of human TRPA1 (e.g., C414S, C421S, C621S, C633S, and C641S) have revealed that these cysteine residues play a critical role in the activation of the channel by electrophilic agonists, which are able to interact with the sulfide moiety of these residues. We, therefore, examined the interaction of 9,10‐PQ with the cysteine residues in TRPA1 using HEK cells transfected with mutant TRPA1 (HEK‐muTRPA1) containing one of the following substitutions: C414S, C421S, C621S, C633S, or C641S. Administration of 9,10‐PQ at a concentration between 0.3 and 10 *μ*mol/L to HEK‐wTRPA1 (Fig. 4A and F) and HEK‐muTRPA1 cells (Fig. 4B–F) induced substantial inward currents at −50 mV, except HEK‐muTRPA1 cells with C414S and C621S substitutions. Even in the presence of higher intracellular Ca^2+^ (3 *μ*mol/L), HEK‐muTRPA1 cells with the C414S substitution were completely insensitive to 9,10‐PQ (Fig. 4F) as well as AITC and Zn^2+^ (Hatano et al. 2013), thus, we excluded this mutant from further analyses. In addition, 9,10‐PQ had little effect on HEK‐muTRPA1 cells with the C621S substitution in Ca^2+^ measuring experiments (Fig. 4G and H). To further confirm the importance of C621 for the 9,10‐PQ‐induced response, we systematically compared the response of wild‐type and mutant TRPA1 with a C621S substitution to 9,10‐PQ and AITC in whole‐cell (Fig. 5A–C) and excised inside‐out patch (Fig. 5D–F) recording configurations. While exposure of HEK‐wTRPA1 cells to both 3 *μ*mol/L 9,10‐PQ and 30 *μ*mol/L AITC induced inward currents and channel activity in both configurations (Fig. 5A, C, D, and F), HEK‐muTRPA1 cells with the C621S substitution responded only to AITC (Fig. 5B, C, E, and F), suggesting that C621 is more important for the response to 9,10‐PQ than AITC. On the other hand, although a quinone‐related compound, 1,2‐naphthoquinone (1,2‐NQ), can activate TRPV1 (Kikuno et al. 2006), 10 *μ*mol/L 9,10‐PQ had no effect on human TRPV1 (Fig. 5G and H).

**Figure 6:**
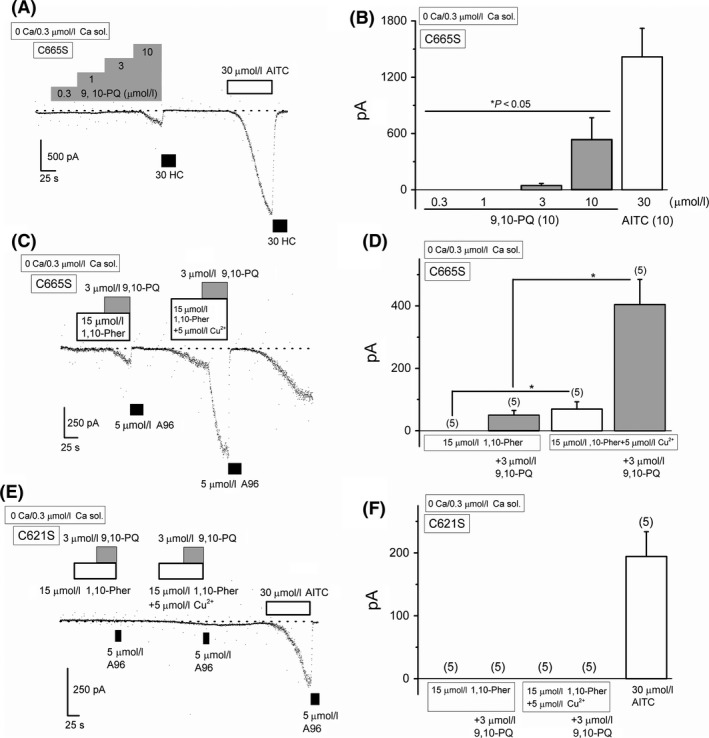
C665 involvement in 9,10‐PQ‐induced TRPA1 channel response. Cells were superfused with 0 Ca SBS and dialyzed with Cs‐aspartate‐rich pipette solution including 0.3 *μ*mol/L Ca^2+^. Ramp waveform voltage pulses from −150 to +50 mV for 100 msec were applied every 5 sec. 9,10‐PQ was commutatively applied to HEK‐muTRPA1 cells with a C665S substitution (C665S) to examine the effects on membrane currents at −50 mV (A) and the pooled data of the peak currents evoked were summarized (B, 10 independent experiments). After application of 10 *μ*mol/L 9,10‐PQ, 30 *μ*mol/L HC (black rectangles) was added to block TRPA1 channel current components. As a comparison, 30 *μ*mol/L AITC was finally applied. *P <* *0.05 by ANOVA. Effects of 9,10‐PQ on sensitizing TRPA1 in HEK‐muTRPA1 cells with C665S (C665S) (C and D) and with C621S substitutions (C621S) (E and F). The TRPA1 mutants were sensitized by Cu^2+^ plus 1,10‐Pher and then exposed to 9,10‐PQ. As a control, each cell was exposed to 9,10‐PQ only in the presence of 1,10‐Pher without Cu^2+^ at the beginning of each experiment. *P <* *0.05 by paired Student's *t*‐test. Bars = SEM.

A recent observation demonstrates that C621 is necessary for human TRPA1 reactivity to electrophilic compounds, and that C665 is secondary (Bahia et al. 2016). Therefore, we next examined the effects of 9,10‐PQ on HEK‐muTRPA1 cells with a C665S substitution. As shown in Figure 6A, application of 0.3–10 *μ*mol/L 9,10‐PQ to this mutant induced substantial inward currents at −50 mV. However, the current responses were clearly smaller (Fig. 6B), suggesting that C665 affects the 9,10‐PQ‐induced response of TRPA1 even though the critical C621 is conserved. To sensitize this mutant channel, we pretreated the C665S HEK‐muTRPA1 cells with 5 *μ*mol/L Cu^2+^ plus 15 *μ*mol/L 1,10‐Pher and then added 3 *μ*mol/L 9,10‐PQ. As a control, we first exposed the mutant to 9,10‐PQ only in the presence of 1,10‐Pher. This Cu^2+^ plus 1,10‐Pher‐induced sensitization recovered the response to 9,10‐PQ in C665S HEK‐muTRPA1 cells (Fig. 6C and D), while not in the C621S HEK‐muTRPA1 cells (Fig. 6E and F), suggesting that C621 is critical and that C665 is supportive for the 9,10‐PQ‐induced response.

**Figure 7:**
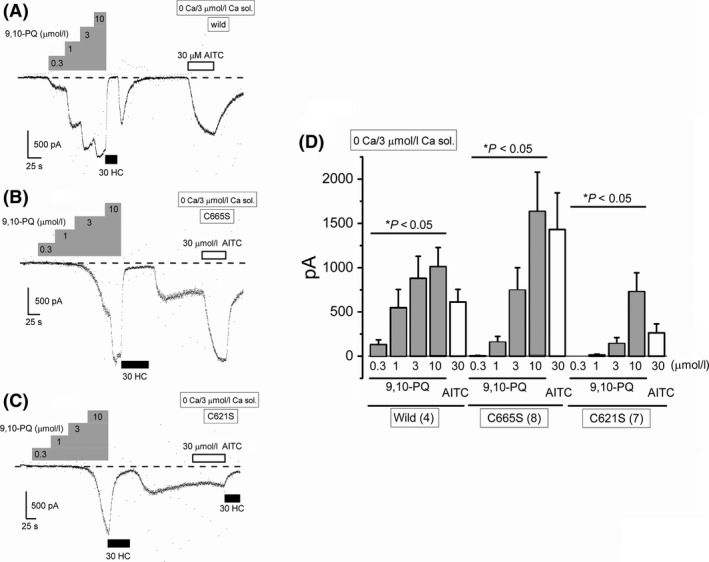
Effects of 9,10‐PQ on TRPA1 sensitization in HEK‐muTRPA1 cells with C665S and C621S substitutions. TRPA1 channels were sensitized by intracellular dialysis of 3 *μ*mol/L Ca^2+^. Cells were superfused with 0 Ca SBS. Ramp waveform voltage pulses from −150 to +50 mV for 100 ms were applied every 5 sec. 9,10‐PQ was commutatively applied to HEK‐wTRPA1 (WT) cells (A, four independent experiments), HEK‐muTRPA1 cells with a C665S substitution (C665S) (B, eight independent experiments), and HEK‐muTRPA1 cells with a C621S substitution (C621S) (C, seven independent experiments) to examine the effects on membrane currents at −50 mV, and the pooled data of the peak currents evoked were summarized (D). After application of 10 *μ*mol/L 9,10‐PQ, 30 *μ*mol/L HC was added to block TRPA1 channel current components. As a comparison, 30 *μ*mol/L AITC was applied. *P <* *0.05 by ANOVA. Bars = SEM.

**Figure 8:**
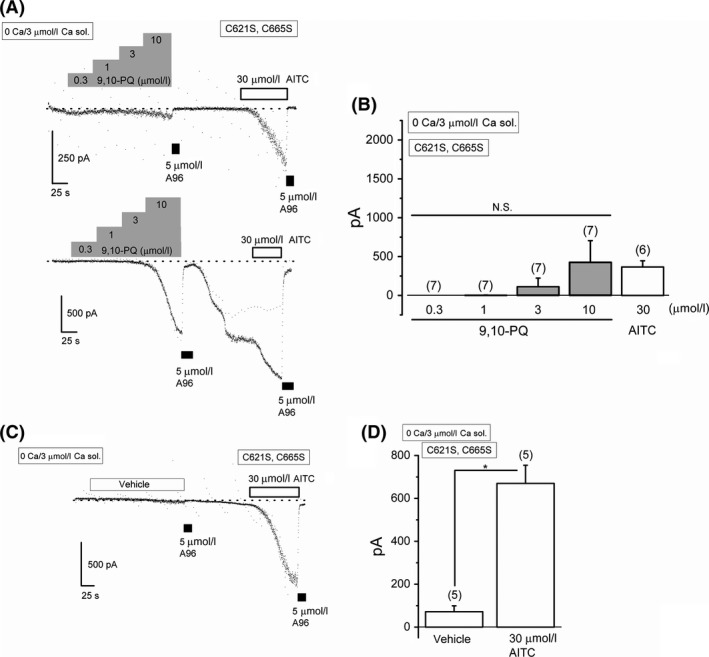
Effects of 9,10‐PQ on TRPA1 sensitization with the double C665S and C621S mutation. TRPA1 channels were sensitized by intracellular dialysis of 3 *μ*mol/L Ca^2+^. Cells were superfused with 0 Ca SBS. Ramp waveform voltage pulses from −150 to +50 mV for 100 msec were applied every 5 sec. (A) and (B) 9,10‐PQ was commutatively applied to HEK‐muTRPA1 cells with the double mutation (C621S, C665S) (A, 6–7 independent experiments) to examine the effects on membrane currents at −50 mV. Two representative current traces are shown (A). The pooled data of the peak currents evoked were summarized (B). After application of 10 *μ*mol/L 9,10‐PQ, 5 *μ*mol/L A96 (black rectangles) was added to block TRPA1 channel current components. As a comparison, 30 *μ*mol/L AITC was applied. (C) and (D) Time‐matched control experiments superfused with the vehicle (DMSO) were performed using the double mutant. After exposure to the vehicle, 30 *μ*mol/L AITC was applied, and vehicle‐ and AITC‐evoked currents were summarized (D, 5 independent experiments). *P <* *0.05 by paired Student's *t*‐test. Bars = SEM.

It is well known that intracellular dialysis of high Ca^2+^ sensitizes TRPA1 channel activity (Wang et al. 2008). Therefore, we internally applied 3 *μ*mol/L Ca^2+^ to both C621S and C665S HEK‐muTRPA1 cells and examined the effects of 9,10‐PQ on the membrane currents of these cells at −50 mV. Similar to wild‐type TRPA1 (Fig. 7A), the mutant TRPA1 with the C665S substitution had a substantial response to 9,10‐PQ (Fig. 7B and D). Although the mutant TRPA1 with the C621S substitution also responded to 9,10‐PQ, the response was relatively small (Fig. 7C and D); hence, we next examined the effects of 9,10‐PQ on a double mutant TRPA1 where both C621 and C665 were replaced with serine. When this mutant TRPA1 was sensitized by 3 *μ*mol/L Ca^2+^, 0.3–10 *μ*mol/L 9,10‐PQ induced A96‐sensitive small inward currents less than −70 pA at −50 mV in five of seven cells (29.7 ± 14.1 pA, upper trace in Fig. 8A). In two cells, however, 10 *μ*mol/L 9,10‐PQ induced A96‐sensitive inward currents larger than −900 pA (1422 ± 484 pA, lower trace in Fig. 8A). In contrast, the vehicle‐induced inward currents at −50 mV were negligible under the same experimental conditions (Fig. 8C and D). Taken together, at relatively low concentrations 9,10‐PQ mainly targets TRPA1 C621 and at higher concentrations 9,10‐PQ may have additional effects on sensitizing TRPA1 even without C621. On the other hand, although C665 plays an important role in 9,10‐PQ induced activation of TRPA1, it is unlikely that C665 is a target for 9,10‐PQ.

**Figure 9:**
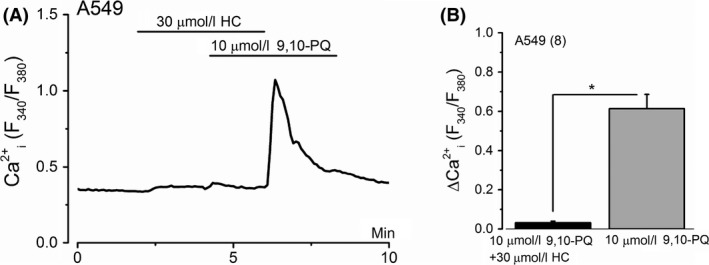
Application of 9,10‐PQ induces a Ca^2+^ response in human alveolar A549 cells. (A) Ca^2+^ response of a representative cell to 9,10‐PQ (10 *μ*mol/L) with or without HC (30 *μ*mol/L). (B) The peak change in Ca^2+^ response was summarized with or without HC (eight independent experiments). *P <* *0.05 by paired Student's *t*‐test. Bars = SEM.

Since 9,10‐PQ is commonly inhaled in the air, airway organs are potential primary targets of 9,10‐PQ. Therefore, we used human alveolar A549 cells to examine whether 9,10‐PQ can activate endogenous TRPA1 expressed in human cells. As shown in Figure 9A, application of 10 *μ*mol/L 9,10‐PQ to A549 cells evoked a Ca^2+^ response in the absence of HC. Since the response was effectively inhibited by preadministration of 30 *μ*mol/L HC (Fig. 9B), A549 cells express TRPA1 which can be a potential target of 9,10‐PQ acutely administrated.

## Discussion

In the present study, we show that the environmental pollutant 9,10‐PQ potently activates human TRPA1 and might function as an irritant when inhaled. In addition, the activation of TRPA1 by 9,10‐PQ is dependent on the cysteine residues at 621 and 665 in the N‐terminus of the channel. Application of 9,10‐PQ also induced activation of endogenous TRPA1 in A549 cells derived from human alveoli, suggesting that environmental 9,10‐PQ may cause acute biological actions via activation of TRPA1.

In this study, we clearly showed that 9,10‐PQ, an environmental pollutant included in cigarette smoke extract (CSE) and diesel exhaust particles (DEP), is an effective activator of human TRPA1. Under all experimental conditions, 1 *μ*mol/L 9,10‐PQ sufficiently activated wild‐type human TRPA1. Indeed, CSE, DEP, wood smoke particulate material (WSPM), and insoluble coal fly ash particles can activate TRPA1 (Andre et al. 2008; Deering‐Rice et al. 2011, 2015; Shapiro et al. 2013), and some putative active chemicals composed of these components were identified: crotonaldehyde and acrolein in CSE; ditert‐butylphenols in DEP; and agathic acid, coniferaldehyde, and perinaphthenone in WSPM. Moreover, additional TRPA1 agonistic compounds might be generated in the lung from polycyclic aromatic hydrocarbons inhaled via cytochrome P450 (P450) (Lanosa et al. 2010). Among these chemicals, acrolein was relatively more potent and its EC_50_ for the activation of rat and human TRPA1 expressed in HEK cells was 0.8–5 *μ*mol/L (Bautista et al. 2006; Andre et al. 2008). In our recent study, 9,10‐PQ was the most potent activator among 1395 CSE components using an antioxidant response element assay in human bronchial epithelial BEAS‐2B cells and, furthermore, 9,10‐PQ was 12.6 times more effective than acrolein in this assay (Sekine et al. 2016). Although it is difficult to measure the exact amounts of these chemicals in CSE and DEP, 9,10‐PQ can be one of the most important chemicals in pollutants as an irritant that affects human health.

Among the TRPA1 agonists employed in this study, 9,10‐PQ was the most potent, although accurate comparison of agonistic activity among chemicals is difficult. As shown in Figure 1, inclusion of Ca^2+^ in the bathing solution caused potentiation and desensitization of 9,10‐PQ‐induced TRPA1 channel currents, as previously reported (Doerner et al. 2007; Zurborg et al. 2007; Wang et al. 2008). It is, therefore, notable that using pharmacological assays to measure TRPA1 agonist‐induced Ca^2+^ may have limitations. On the other hand, TRPA1 whole‐cell currents were sustainably and cumulatively activated by application of 9,10‐PQ and AITC in the absence of extracellular Ca^2+^, suggesting that these are direct activators of human TRPA1 with little dependency on Ca^2+^, and 9,10‐PQ is more potent than AITC. In contrast, under the same experimental conditions, the agonistic activities of Zn^2+^ and Men were weak and negligible, respectively. Since inclusion of 3 *μ*mol/L Ca^2+^ in the pipette solution increased the response to 10 *μ*mol/L Zn^2+^ (52.0 ± 5.2 pA, *n* = 3 vs. 339.7 ± 24.9 pA, *n* = 4 in the presence of 0.3 and 3 *μ*mol/L Ca^2+^, respectively, at −50 mV) and 300 *μ*mol/L Men (6.25 ± 6.25 pA, *n* = 4 vs. 339.7 ± 24.9 pA, *n* = 4 in the presence of 0.3 and 3 *μ*mol/L Ca^2+^, respectively, at −50 mV), Zn^2+^ and Men could be TRPA1 agonists in a Ca^2+^‐dependent manner. In fact, 1 *μ*mol/L Zn^2+^ and 250 *μ*mol/L Men are substantially effective in the elevation of intracellular Ca^2+^ in HEK‐wTRPA1 cells (Xiao et al. 2008; Hu et al. 2009).

The quinone moiety of 9,10‐PQ is crucial for the activation of TRPA1 because Phenan, in which the quinone moiety was removed from 9,10‐PQ, had little effect on membrane currents in HEK‐wTRPA1 cells (Fig. 3G). Moreover, 1,10‐P‐5,6‐D, which has a quinone moiety and similar structure to 9,10‐PQ, also activated the TRPA1 currents that were sensitive to HC. However, the potency of 1,10‐P‐5,6‐D was weak in comparison to 9,10‐PQ (Fig. 3F and H), suggesting that the main structure of chemicals affects electrophile‐induced binding to channel cysteines and subsequent activation. Consistently, para‐benzoquinone activates TRPA1 more potently than 9,10‐PQ, though the activation is independent of the cysteines at 621 and 665 (Ibarra and Blair 2013).

Although superoxide and hydroxyl radicals are generated by enzymatic one‐electron reduction of 9,10‐PQ by P450 reductase within 30 sec of treatment (Sugimoto et al. 2005), it is unlikely that intracellular factors are involved in 9,10‐PQ‐induced activation of TRPA1. Based on the evidence that application of 9,10‐PQ to outside‐out (Fig. 2) and inside‐out patches (Fig. 5D) excised from HEK‐wTRPA1 cells effectively induced HC‐sensitive channel activity, we concluded that a membrane‐delimited pathway must be involved in the activation. On the other hand, it has been shown that polyphosphates are required for electrophilic channel activity, which stabilizes the coiled‐coil domains in the intracellular C‐terminus (Kim and Cavanaugh 2007; Paulsen et al. 2015). Since 9,10‐PQ induced TRPA1 channel activity without TPP in excised outside‐out patches (Fig. 2D), this suggests that polyphosphates are not essential for channel activation. Nevertheless, it is evident that TPP stabilizes (Fig. 1E and F) and potentiates (Fig. 1H and I) 9,10‐PQ‐induced responses.

Using C621S and C665S mutants, we found that C621 is critical but not sufficient for channel activation by 9,10‐PQ in nonsensitizing cells. In fact, TRPA1 with a C665S substitution had a substantially smaller response to 9,10‐PQ (Fig. 6B), demonstrating a requirement for C665 in the activation. Since sensitization of the C665S mutant by Cu^2+^ plus 1,10‐Pher and higher intracellular Ca^2+^ reversed the response, it is plausible that C665 may play an obligatory role in TRPA1 activation downstream of covalent modification of C621 by 9,10‐PQ. In contrast, responses of the C621S mutant to 9,10‐PQ were not restored by Cu^2+^ plus 1,10‐Pher sensitization, and were, in part, reversed by higher intracellular Ca^2+^, confirming that the high reactivity of C621 to 9,10‐PQ is critical for channel activation. Consistently, it has been shown that the exceptionally high reactivity of C621 in TRPA1 contributes to channel activation by electrophiles (Bahia et al. 2016). On the other hand, double mutants with C621S and C665S substitutions sensitized by higher intracellular Ca^2+^ did not significantly respond to 9,10‐PQ, while two cells had a clear current activation by 10 *μ*mol/L 9,10‐PQ. The sensitized TRPA1 channel may be directly activated by 9,10‐PQ via other cysteine residues and lysine residues at 620 and/or 708 (Hinman et al. 2006; Bahia et al. 2016).

The physiological significance of TRPA1 is still unclear in human non‐neuronal cells, although keratinocytes, bronchial epithelial cells, and inflammatory synoviocytes express the channel (Atoyan et al. 2009; Mukhopadhyay et al. 2011; Hatano et al. 2012). In the present study, we identified that the air pollutant 9,10‐PQ as an effective activator of human TRPA1. Since 9,10‐PQ is included in CSE and DEP, it is likely that 9,10‐PQ acutely activates TRPA1 in airway organs when inhaled. Moreover, CSE upregulates expression of TRPA1 in A549 cells (Nie et al. 2016). However, the limitation of this study is that the final concentration of 9,10‐PQ around airway organs has not been determined when exposed to cigarette smoke and pollutant air. Nevertheless, a metabolite of 9,10‐PQ was found in the urine of both smokers and nonsmokers, suggesting that 9,10‐PQ is commonly taken into the human body (Asahi et al. 2014). Taken together, inhaled 9,10‐PQ acutely and directly could activate human TRPA1, and may have some pathophysiological influence. In fact, acrolein in CSE contracted guinea pig tracheal smooth muscle via a TRPA1‐mediated pathway (Andre et al. 2008). In contrast, acrolein caused TRPA1‐dependent tracheal relaxation in mouse (Cheah et al. 2014).

In conclusion, the air pollutant 9,10‐PQ can directly activate TRPA1; the activation is dependent on the channel's C621 residue, and is, in part, also regulated by the function of its C665 residue. Moreover, the quinone moiety of 9,10‐PQ is essential for activation. Since 9,10‐PQ is included in CSE and DEP, and is easily inhaled, 9,10‐PQ may be an important irritant in airway organs.

## Author Contributions

K. Muraki participated in research design. K. Muraki, Y. Ando, H. Suzuki, and Y. Muraki conducted experiments. T. Sekine, T. Hirata, and N. Hatano contributed new reagents or analytic tools. K. Muraki and T. Sekine wrote or contributed to the writing of the manuscript.

## Disclosure

None declared.
